# Combinatorial effects of telmisartan and docetaxel on cell viability and metastatic gene expression in human prostate and breast cancer cells

**DOI:** 10.22099/mbrc.2022.42638.1700

**Published:** 2022-03

**Authors:** Marjan Khorsand, Zohreh Mostafavi-Pour, Vahid Razban, Sahar Khajeh, Razieh Zare

**Affiliations:** 1 Department of Biochemistry, School of Medicine, Shiraz University of Medical Sciences, Shiraz, Iran; 2 Student Research Committee, Shiraz University of Medical Sciences, Shiraz, Iran; 3 Autophagy Research Center, Shiraz University of Medical Sciences, Shiraz, Iran; 4 Molecular Medicine Department, School of Advanced Medical Sciences and Technology, Shiraz University of Medical Sciences, Shiraz, Iran; 5 Stem Cell Technology Research Center, Shiraz University of Medical Sciences, Shiraz, Iran; 6 Bone and Joint Diseases Research Center, Shiraz University of Medical Sciences, Shiraz, Iran; 7 ^#^Zohreh Mostafavi-Pour and Vahid Razban are both corresponding authors and have got the same contribution in this work

**Keywords:** Cancer, Combination index, EMT, Slug, Snail

## Abstract

The epithelial-to-mesenchymal transition (EMT) is a unique process resulting in enhanced cell motility, invasiveness, and metastasis in cancer. The EMT is regulated by several transcription factors, including Snail and Slug, which exert crucial roles during cancer progression. We have studied the effects of Docetaxel as the first-line chemotherapy agent for prostate cancer, and Telmisartan as an anti-hypertensive drug on the expression level of Snail and Slug. In addition, the effects of Docetaxel, Telmisartan and their combination on cancer cell proliferation were investigated. The PC3, DU145, MDA-MB468, and HEK cell lines were used for this study. Quantitative RT-PCR analysis and MTT assay were used to study the expression of Snail and Slug level and cell proliferative assay, respectively. We found that a combination of Docetaxel + Telmisartan effectively inhibits the cell proliferation in cancerous cells in comparison with each drug alone (P<0.05). Furthermore, in these cell lines, Docetaxel, Telmisartan and their combination significantly diminished the expression level of Snail and Slug genes compared to control cells (P<0.001), however, in the HEK cell line, this effect was seen only in the combination group. Our data imply that Telmisartan and its combination with Docetaxel exert strong inhibitory effects on the expression level of Snail and Slug genes. Also, these drugs and their combination could inhibit cancer cell proliferation. In conclusion, the combination of Telmisartan and Docetaxel has the potential to suppress the metastasis of prostate and breast cancer cells.

## INTRODUCTION

Cancer is the second leading cause of death and remains a source of increasing concern globally. Prostate cancer is the most prevalent malignancy in men worldwide that could be treated by several approaches including, prostatectomy, radiotherapy, and chemotherapy at the early stage [1]. Each of these approaches has various side effects such as, nausea, diarrhea, pain, skin erythema, and damage to surrounding normal tissues [2]. Prostate cancer is a hormone-dependent tumor in which the androgen receptor and its signaling pathway were altered. Despite the treatments that are given for patients, the tumor has ultimately developed resistance to androgen deprivation therapy in the majority of advanced prostate cancer patients that is annotated castration-resistant prostate cancer (CRPC) [3].

The metastasis process in cancer involves multiple steps. In the initial stage, the cancerous cells detach from the primary tumor site and migrate to nearby blood or lymph vessels. In later stages, these cells migrate to distant places, reside there and proliferate. A crucial point in the metastases process attributes to the epithelial-to–mesenchymal transition (EMT). EMT is a unique programmed process by which epithelial cells achieve a mesenchymal phenotype. During EMT, the cancer cells deplete their polarity, and adhesion capability and acquire the migration ability as well as the invasive potential to other tissues. It is well known that EMT has a vital role in cancer progression and metastases [4].

More specifically, the EMT process demonstrated various hallmarks, such as E-cadherin down-regulation, which is associated with decreasing in cell-cell adhesion, up-regulation of matrix metalloproteinase (MMPs), and high expression of mesenchymal-related proteins including Vimentin and N-cadherin to increase cell motility. Furthermore, a series of EMT inducers have been described, such as β-catenin, and the Snail family members [5]. The Snail superfamily contains three proteins including, Snail (SNAI1), Slug (SNAI2), and Snail3. Snail and Slug had a similar function and crucial roles in various physiological and pathological pathways. Previous studies reported that Snail and Slug genes participated in the progression of several cancers. The Snail and Slug, as the transcriptional repressors of E-cadherin, have essential roles in the induction and development of EMT [6].

Docetaxel (DTX) as a therapeutic agent is commonly used to treat a broad range of human malignancies, including lung cancer, stomach, head and neck, breast, and prostate cancer [7-9]. DTX is an anti-mitotic agent, which prevents microtubule disassembly and has been identified to down-regulate transcription of the androgen receptors. DTX is known as the standard first-line drug for prostate cancer treatment; however, using this chemotherapy agent in a long term, leads to drug resistance and many adverse effects [10]. Therefore, finding new drugs with fewer side effects that can inhibit the migration and metastasis of malignant cells seems to be very valuable.

Today, angiotensin II receptor blockers (ARBs) are widely administrated to treat hypertension, chronic kidney disease, and heart failure. Previous investigations indicated that angiotensin II is correlated with cancer progression, and ARBs could antagonize angiotensin receptors, and inhibit tumor growth. Among these ARBs, Telmisartan (Tel) could inhibit cell proliferation and leads to apoptosis in the different types of cancer cells, including gastric, colon [11], and urological [12, 13] cancers [14]. Because understanding the roles of Snail and Slug provides new insights into the molecular mechanisms of tumor invasion in the EMT process, in the current study the effects of Tel, DTX and their combination on cell viability and the expression of Snail and Slug as EMT specific genes were investigated. The two main types of prostate cancer cell line (PC3 and DU145), a triple-negative breast cancer cell line (MDA-MB468), and a normal human cell line (HEK) were used for the present study. We evaluated the response of each cell line to the aforementioned drugs.

## MATERIALS AND METHODS

**Reagents and materials:** RPMI-1640, fetal bovine serum(FBS), penicillin–streptomycin, and Trypsin/EDTA were obtained from Invitrogen Life Technologies (Gibco, Life Technology, UK). DTX and Tel were purchased from MedChem Express (Princeton, NJ, USA). All drugs were dissolved in dimethyl sulfoxide (DMSO; Sigma-Aldrich, MO, USA) and were diluted with a culture medium before each experiment. All primers were obtained from Metabion International AG (Germany).

**Cell cultures:** The PC3, DU145 (Prostate cancer cell lines), MDA-MB468 (Breast cancer cell line), and HEK (Human Embryonic Kidney cell line) were purchased from the National Cell Bank of Iran (Pasteur Institute, Iran). The cell lines were cultured in RPMI-1640 supplemented with 10% FBS, 100 U/ml of penicillin, and 100 μg/ml of streptomycin, in an incubator with 5% CO_2_ atmosphere at 37˚C. 

**Cell-proliferative assay:** The cells were seeded onto 96 well plate (SPL life sciences, Korea) and incubated in a CO_2_ incubator at 37˚C. After 24h, the cells were treated with DTX and Tel dissolved in DMSO. The final concentration of DMSO was 0.1%. Cell viability was determined after 48h incubation using  3-(4,5-dimethylthiazol-2-thiazolyl)-2,5-diphenyltetrazo-lium bromide (MTT) assay and presented as the percentage of the control group which was treated by medium cell culture [15].

**Analysis of Interactions, Combination Index:** For evaluation the in vitro pharmacological drug interaction for DTX and Tel, combination index (CI) is determined [16]. For this purpose, first, the cells were exposed to each drug alone, DTX (0.001-100 nM) and Tel (0.001-100 µM), and the IC50 values for each cell line were calculated, then the cells were treated with the combination of DTX +Tel. The PC3 and DU145 cell lines were treated with different doses of DTX (0.005-10 nM) +Tel (0.005-10 µM) for 48 h. The MDA-MB 468 cells were treated with different doses of DTX (0.5,1 nM) +Tel (0.1-100 µM), whereas, HEK cells were treated with DTX (0.0005,0.001 nM) + Tel (0.0005,0.001 µM) for 48h. Cell viability was evaluated using an MTT assay, as previously reported [15]. The concentration of drugs for combination treatment was selected based on maximum effect, and IC50 of each drug (data not shown). The resulting combination index (CI=1) indicates an additive effect, synergism (CI<1), and antagonism (CI>1) in drug combinations. 

**RNA isolation and Quantitative Real-Time PCR (qRT-PCR): **Expression of Snail and Slug genes were quantitated by qRT-PCR and Glucuronidase Beta (GUSB) gene was used as internal control gene. Cells were cultured in 25 cm^2^ flask (7×10^5^cells in each flask) and after 24h, the cells were treated with DTX (0.01 nM), Tel (0.1 µM), or their combinations for 48h. Total RNA was isolated from the cells by TriPure isolation reagent (Roche, Germany) as described by the manufacturer’s protocol. After determination of RNA concentration by a Nanodrop 1000 spectrophotometer (NanoDrop Technologies; Thermo Fisher Scientific, Inc. Wilmington, DE, USA), the integrity of RNA was evaluated using gel electrophoresis. To prepare cDNA, a reverse transcriptase kit (Fermentas; Thermo Fisher Scientific, Inc., Pittsburgh, PA, USA) was used and the cDNA was made from 1 μg of total RNA. QRT-PCR was carried out triplicate using SYBR green PCR master mix, and Rotor Gene Q-series Real-Time PCR Systems (QIAGEN, USA). The qPCR process was carried out for 40 cycles using the following protocol: each cycle included 95˚C for 10 min, 95˚C for 15 secs, 58˚C (for GUSB, Slug) or 62.9 (for Snail) for 30 secs, 72˚C for 30 secs and 72˚C for 10 min. The sequences of primers for the determination of Snail, Slug and GUSB genes expression are shown in Table 1. The relative expression of Snail and Slug genes was calculated using the Pfaffl method, as described previously [17].

**Table 1:** List of primers were used for Real-time PCR

**Genes **	**Primer sequence**	**Amplicon size**
Glucuronidase Beta (GUSB)	F: TCGCTCACACCAAATCCTT R: GGCTTCTGATACTTCTTATACCA	205 bp
Snail	F: CCTGCGTCTGCGGAACCTG R: ACATCTGAGTGGGTCTGGAGG	163 bp
Slug	F: AAGGACACATTAGAACTCACA R: CTACACAGCAGCCAGATT	198 bp

**Statistical Analyses: **Data in the present study were derived from at least three independent experiments. Statistical significance among all groups was evaluated using One-way Analysis of Variance (ANOVA) followed by the Tukey test. P-value<0.05 was considered statistically significant. The Real-time PCR graphs were done using GraphPad Prism (Version 6.0), and analysis of Combination Index (CI) and related graphs were done, using CompuSyn software.

## RESULTS

Due to the potential toxicity of DTX, we examined the effect of combination treatment involving DTX+ Tel to reduce the DTX dose that necessary to show its anti-cancer effects. The cells were treated with a combination of DTX +Tel at varying concentrations. Treatment of PC3 cell line with DTX 0.01 nM+Tel 0.01 µM inhibited the cell viability by 55%, whereas DTX and Tel alone by the same concentration inhibited the cell viability by 29% and 32%, respectively (P<0.001). In addition, treatment of these cells with DTX 0.005 nM+Tel 0.005 µM had more inhibitory effect on cell viability compared to DTX 0.01 nM (P<0.05). Treatment of DU145 cells with DTX 0.1 nM+ Tel 0.1 µM inhibited the cell viability by 61%, whereas DTX and Tel alone by the same concentration inhibited the cell viability by 35% and 7%, respectively (P< 0.001). In MDA-MB468 cell line DTX 0.5 nM+ Tel 100 µM and DTX 1 nM+ Tel 100 µM inhibited the cell viability by 55% and 57% respectively, whereas DTX 1 nM diminished the cell viability by 39% (P<0.05).

Our results showed that the use of combination treatment in doses much lower than the drugs IC50, for normal cells (HEK) had significant effect in inhibiting cell growth. So that, treatment of these cells with DTX 0.0005 nM+ Tel 0.001 µM, DTX 0.001 nM+ Tel 0.0005 µM, and DTX 0.001 nM+ Tel 0.001 µM had the significant inhibitory effects on cell viability compared to the Tel 0.001 µM treatment (30%, 45%, 35%, and 0.75% respectively, P<0.001). Also, DTX 0.001 nM+ Tel 0.0005 µM significantly inhibited cell viability compared to the DTX in both doses of 0.0005 nM and 0.001 nM (P<0.05).

Over the past century, many attempts have been made by researchers to quantify the dose-effect relationships of each drug on its own or the combinations to answer the question of whether or not the combination of two drugs can have a synergistic effect. To determine the effects (synergistic, antagonistic, or additive) of the combination treatment on cell lines, we mathematically evaluated the results using Chou and Talalay method [18]. For this reason, the cells were treated with a combination of drugs at a constant ratio (1:1) for PC3, DU145 cells, and a non-constant ratio for MDA-MB468, HEK cells, or drugs alone. Fraction affected (Fa) values, show the fraction of cells that inhibited after drug treatment, and Fu values, show the fraction that unaffected after drug treatment. These values were obtained from cells in different drug concentrations. Median effect plot was used for evaluation of pharmacodynamics median doses for toxicity (TD50), lethality (LD50), effect of agonist drugs (ED50), and effect of antagonist drugs (IC50). To show the results at different Fa values, combination index (CI) values were determined for each Fa and the FA‑CI plot was constructed. In the different treatments, two concentrations that used for PC3 cells and three concentrations that used for other cell lines, CI values were below 1.0 (Table 2). However, in some concentrations, combination treatment elevated CI value to more than 1.0. The Dose-Reduction Index (DRI) is a mathematical index that shows how many folds each drug dose in a synergistic combination may be decreased, at a certain effect level, in comparison with the dose of each drug separately. In the present study, this index was measured for binary drug combination and DRI=1 demonstrates no dose reduction, whereas DRI>1 and <1 show favorable and unfavorable dose-reduction, respectively. In PC3 cell line, at Fa=0.46, DRI was 17.8 and 36.6 for DTX and Tel, respectively. Similarly, at Fa=0.61, DRI was calculated 3 and 5.4 for DTX and Tel, respectively. The parameters obtained from other cell lines are also presented in Table 2. In addition, the more data and plots about combination treatment are presented in the supplementary files (Fig. S1, Fig. S2, and Fig. S3). 

**Table 2:** Data summary of dose-effect curves and Chou-Talalay method parameters of drug combinations against prostate cancer cell lines, MDA-MB468, and HEK cell lines after 48 h treatment period

**Cell line**	**Fa**	**CI**	**DRI DTX**	**DRI Tel**	
**PC3**	0.46	0.08	17.8	36.6	
	0.61	0.5	3.09	5.4
				
**DU145**	0.39	0.001	1248.8	1444.4	
	0.47	0.09	11.9	121.3
	0.54	0.11	8.8	577
				
**MDA-MB468**	0.43	0.018	260	66.3	
	0.45	0.038	270	28.7
	0.55	0.31	10	4.6
				
**HEK**	0.55	0.002	401	197346	
	0.65	0.09	10.7	37446
	0.7	0.33	2.9	22071	

Fa; Fraction inhibition, CI; Combination index, DRI; Dose-Reduction Index.

**Figure 1:**
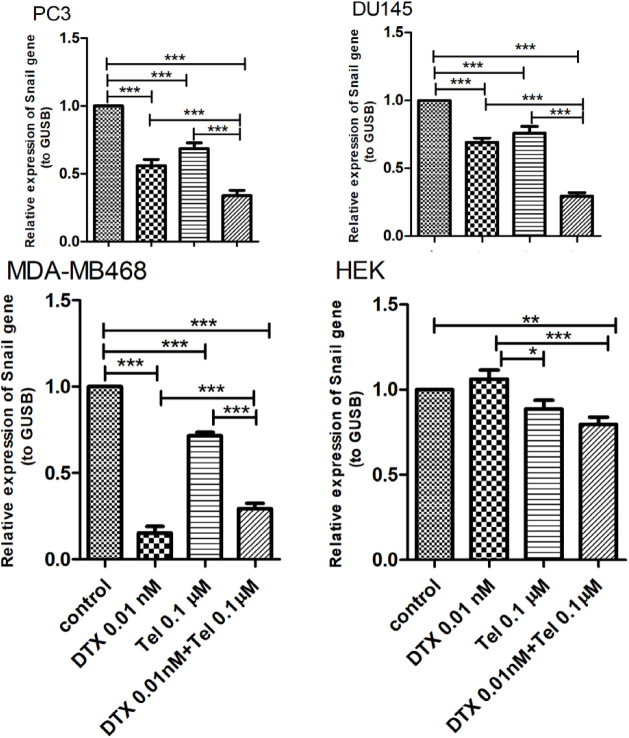
The effect of combination treatment with DTX and Tel on Snail mRNA expression in cancer cell lines (PC3, DU145, MDA-MB468) and HEK cell line. The graph shows the percentage of relative expression of Snail gene in the treated groups to compare with the control (mean ± standard deviation, n=6). DTX; Docetaxel, Tel; Telmisartan

In this study the expression level of Snail mRNA was also performed using the quantitative reverse transcription PCR (RT-qPCR). As shown in Figure 1, in the PC3 cell line, Snail mRNA expression decreased down 44% after treatment with DTX (P<0.001), 32% after treatment with Tel (P<0.001) and 66% (P<0.001) after combination treatment compared to the untreated control group. In the DU145 cell line, the expression level of Snail decreased by 31% in DTX (P<0.001), 24 % in Tel (P<0.001) and 71% (P<0.001) in combination groups compared to the untreated control group. In the MDA-MB468 cell line also DTX, Tel, and DTX+Tel treatment resulted in a significant reduction in mRNA expression of Snail in comparison with the untreated control group (P<0.001). Also, treatment of these three cell lines with DTX+Tel resulted in a significant reduction in Snail mRNA expression compared to the treatment with each drug separately (P<0.001). However, in the HEK cell line, relative changes in Snail mRNA expression in DTX and Tel groups were not significant, yet in the combination group, it decreased by 21% (P<0.01).

**Figure 2:**
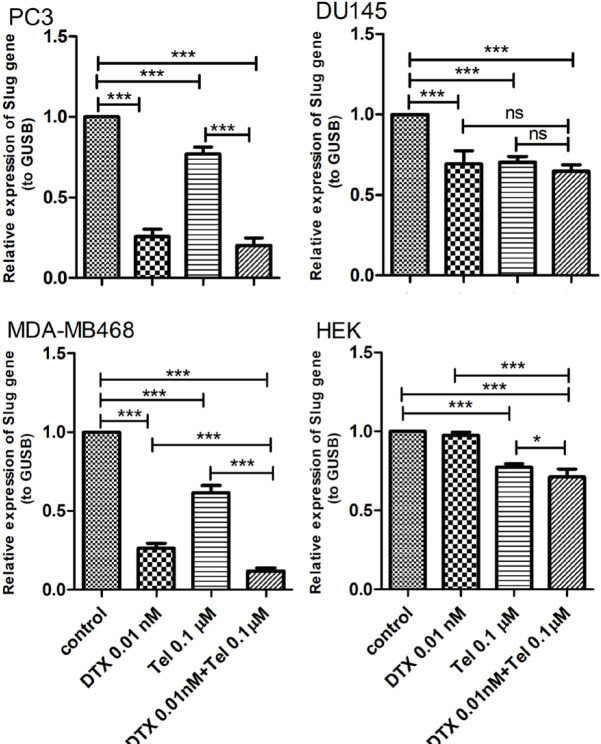
The effect of combination treatment with DTX and Tel on Slug mRNA expression in cancer cell lines (PC3, DU145, MDA-MB468) and HEK cell line. The graph shows the percentage of relative expression of Slug gene in the treated groups to compare with the control (mean ± standard deviation, n=6). DTX; Docetaxel, Tel; Telmisartan

As shown in Figure 2, RT-qPCR results demonstrated that in PC3 cell line, the expression level of Slug decreased down 75% after treatment with DTX (P<0.001), 23% after treatment with Tel (P<0.001) and 80% (P<0.001) after combination treatment compared to the untreated control group. In addition, the expression level of Slug in the DTX+Tel group was significantly lower than that of the Tel group in these cells (P<0.001). In DU145 cell line, the expression level of Slug decreased by 31% in DTX (P<0.001), 30% in Tel (P<0.001) and 36% (P<0.001) in the combination groups compared to the untreated control group. In the MDA-MB468 cell line, the expression level of Slug decreased down 74 % after treatment with DTX (P<0.001), 39% after treatment with Tel (P<0.001) and 88% (P<0.001) after combination treatment compared to the untreated control group. Also, treatment of these cell lines with DTX+Tel resulted in a significant reduction in Slug mRNA expression compared to each drug separately (P<0.001). However, in the HEK cell line, relative changes in Slug mRNA expression in DTX and Tel groups were not significant, yet in the combination group, it decreased by 29% (P<0.01).

## DISCUSSION

Prostate and breast cancers are the two types of malignancy in humans with the poor prognosis of their patients and high rate of mortality. The conventional chemotherapeutic agents, including Taxanes are still the gold standard among prostate and breast cancer treatment during the early and late stages [19]. However, despite efforts to improve the clinical results of chemotherapy, after lung cancer, breast and prostate cancers in women and men have the highest number of deaths due to cancer in the United States [20]. Since the metastasis to distant organs is the leading cause of death in cancer, finding drugs with minimal side effects that can inhibit the metastasis process is very helpful. For this purpose, we selected two prostate cancer cell lines, PC3 and DU145, and a breast cancer cell line, MDA-MB468, to perform our investigation. In addition, we applied HEK cell line isolated from the human embryonic kidney to explore the potential cytotoxicity of Tel to normal cells as well. 

In the present work, we surveyed the effects of combination treatment of DTX+Tel on prostate, breast cancer, and the HEK cell proliferation. According to our knowledge, this study is the first study about the anti-cancer and anti-metastatic effects of the combination treatment of Tel and DTX on cancer cells.

The anti-proliferative effects of DTX and Tel via a dose-dependent manner were seen in these cell lines. However, the Tel IC50 value in the HEK cell line was much higher than that of cancer cell lines, which shows the toxicity of Tel on the HEK cell line as a normal cell line is remarkably lower than that of cancer cells. An important finding of our study is the significant effect of Tel in reducing the dose of DTX for inducing its anti-cancer effects. Overall, our results showed that combination treatment ameliorated the cytotoxic profile of DTX in PC3, DU145, MDA-MB468, and HEK cell lines compared with the drugs treatment alone.

The results showed that the combination treatment in cancer cell lines was more effective than the drugs alone treatment. In some treatments that used for all cell lines CI values were below 1.0, which indicate the synergistic effects between two drugs at lower doses (Table 2). However, in some treatments, combination treatment elevated CI value to more than 1.0 which indicate an antagonistic effect. In addition, in our study at Fa= 0.46, DRI was 17.8 and 36.6 for DTX and Tel, respectively indicating combination treatment of PC3 cells could reduce DTX dose up to 17.8 times while decreasing Tel dose up to 36.6 times. Similarly, at Fa= 0.61, DRI was calculated 3 and 5.4 for DTX and Tel, respectively. Also, as seen in Table 2 in other cell lines DRI values were more than 1.0, which indicate the favorable dose-reduction in these cell lines.

Many studies have shown that the combination of DTX with some agents and drugs has reduced the clinical dose of DTX. Mansour *et al.* reported that stearidonic acid (an omega-3 fatty acid) could synergistically enhance the cytotoxic effects of DTX in the PC3 and DU145 cells [21]. Treatment of PC3 cells with the combination of DTX and mTOR inhibitor, Temsirolimus, caused a significant reduction in the cell proliferation in comparison with monotherapy by a higher dose of DTX [22]. In agreement with our results, Matsuyama and colleagues showed Tel could inhibit the urological cancer cells growth in a dose-dependent manner. Furthermore, Tel induced DNA fragmentation and early apoptosis in urological cancer cells. However, the other ARBs, such as Candesartan and Valsartan did not decrease the cell proliferation in these cell lines. They suggested that Tel may inhibit cell proliferation in urological cancer cells via the PPAR-γ pathway[23]. It has been shown that Tel is a partial agonist for PPAR-γ and the PPAR-γ expression levels in cancerous cells and tissues were higher than their normal counterparts. Therefore, the use of its ligands (Tel) induces apoptosis in cancer cells and this effect is more than normal cells [23, 24]. Uemura* et al *[25]. reported that administration of Candesartan (a type of ARB) for prostate cancer patients with hypertension caused a significant reduction in the prostate-specific antigen level and improved the cancer outcome in these patients. Matsui *et al.* demonstrated that Tel, via inducing S-phase arrest and reduction in expression of cyclin A2 and cyclin-dependent kinase 2 suppressed the proliferation of Esophageal Squamous Cell Carcinoma cell lines. Additionally, they reported that Tel inhibited tumor growth *in vivo* in a xenograft mouse model [26]. Tel suppressed the proliferation of the human gastric cancer cells *in vivo* and *in vitro* by promotion cell cycle arrest at the G0/G1 phase and inhibiting the phosphorylation of the tumor suppressor retinoblastoma (pRb) protein [27]. Green and colleagues showed cyclic peptide antibiotic Actinomycin D+ Tel treatment reduced *in vitro* lung cancer cell viability and decreased stemness. Furthermore, intratumoral administration of Actinomycin D+ Tel synergistically reduced tumor burden in xenograft mouse models compared to either drug alone. Tel via increasing the drug permeability in tumors has anti-cancer stem cell activity [28]. Tel significantly decreased the growth and migration of the A549 cells, a lung cancer cell line, in a dose and timedependent manner. Besides, treatment of these cells with Tel leads to a reduction in the expression of antiapoptotic protein Bcell lymphoma and elevation in the expression of proapoptotic proteins, including Bclassociated X and caspase3 proteins [29].

The results of the present study also showed, treatment of cancer cell lines, PC3, DU145, and MDA-MB468, with DTX (0.01 nM), Tel (0.1 µM), and their combination caused a significant reduction in the expression level of Snail and Slug mRNA compared to control group. The different outcome was achieved when HEK cells were treated with DTX (0.01 nM), Tel (0.1 µM), and their combination. Snail and Slug gene expression were diminished substantially in the combination group; however, the expression of these genes in monotherapy groups did not make significant difference in comparison with the control group. This finding indicated that Tel and DTX did not have significant effects on normal cells. To the best of our knowledge, the effects of Tel and its combination with DTX on the expression level of Snail and Slug (two transcription factors involved in cancer metastasis) in prostate and breast cancer cells have not been investigated yet. Liu *et al.* demonstrated Tel as an agonist for PPAR-γ, can alleviate the EMT induced by oxalate and calcium oxalate crystals via expending antioxidant effects through the PPAR-γ-AKT/STAT3/p38 MAPK-Snail signaling pathway. Snail as a regulator of E-cadherin could bind to E-box elements within E-cadherin promoter and suppress the transcription of E-cadherin that finally leads to the loss of cell-cell adhesion in epithelial cells and increases the EMT development [30, 31]. In another study, Emadi Baygi *et al.* showed the expression of Slug mRNA mainly was at a high level in the PC3 cell line and downregulation of Slug, using specific siRNA induced a significant increase in E-cadherin expression and decreasing in cell migration through a matrigel matrix. Also, the cell viability is significantly diminished after Slug suppression in the PC3 cell line. Overexpression of either Snail or Slug is sufficient for EMT induction and progression in cultured epithelial cells [32]. Therefore, inhibition of these genes using drugs other than chemotherapy drugs can greatly help to reduce cancer cell invasion.

In conclusion, the results of our study showed that combination treatment of prostate and breast cancer cells with DTX+Tel significantly decreased cell viability. Using combination therapy to treat various cancers may help to diminish the administrated dose of chemotherapeutic agents and subsequently reduce the side effects of these drugs. Furthermore, a subsequent decrease in Snail and Slug genes expression was noted in PC3, DU145, and MDA-MB468 cells. However, these results were slightly different for HEK cells as a normal cell line after treatment with Tel and DTX in terms of Snail and Slug gene expression. Since increased Snail and Slug genes expression, as master proteins for EMT regulation, have an essential role in tumor metastasis, finding drugs with the ability to suppress the expression of these genes with minimum side effects can be considered as an important strategy for decreasing the EMT process and cancer cell invasion and migration. 

However, future *in vivo* studies and randomized clinical trials are required to clarify its mechanism to establish the strategy of combination therapy for prostate and breast cancers.

## Conflict of Interest

The authors report no conflict of interest.

## References

[1] Lu X, Chen D, Yang F, Xing N (2020). Quercetin inhibits epithelial-to-mesenchymal transition (EMT) process and promotes apoptosis in prostate cancer via downregulating lncRNA MALAT1. Cancer Manag Res.

[2] Afkham A, Aghebati-Maleki L, Siahmansouri H, Sadreddini S, Ahmadi M, Dolati S, Afkham NM, Akbarzadeh P, Jadidi-Niaragh F, Younesi V, Yousefi M (2018). Chitosan (CMD)-mediated co-delivery of SN38 and Snail-specific siRNA as a useful anticancer approach against prostate cancer. Pharmacol Rep..

[3] Mullane SA, Van Allen EM (2016). Precision medicine for advanced prostate cancer. Curr Opin Urol.

[4] Parol M, Gzil A, Bodnar M, Grzanka D (2021). Systematic review and meta-analysis of the prognostic significance of microRNAs related to metastatic and EMT process among prostate cancer patients. J Transl Med.

[5] Fontana F, Raimondi M, Marzagalli M, Sommariva M, Limonta P, Gagliano N (2019). Epithelial-to-mesenchymal transition markers and CD44 isoforms are differently expressed in 2D and 3D cell cultures of prostate cancer cells. Cells.

[6] Yu H, Shen Y, Hong J, Xia Q, Zhou F, Liu X (2015). The contribution of TGF-β in Epithelial–Mesenchymal Transition (EMT): Down-regulation of E-cadherin via snail. Neoplasma.

[7] Banerjee S, Singh SK, Chowdhury I, Lillard Jr JW, Singh R (2017). Combinatorial effect of curcumin with docetaxel modulates apoptotic and cell survival molecules in prostate cancer. Front Biosci (Elite Ed).

[8] Hoffman A, Sasaki H, Roberto D, Mayer MJ, Klotz LH, Venkateswaran V (2016). Effect of Combination therapy of Desmopressin and Docetaxel on prostate cancer cell (DU145) proliferation, migration and tumor growth. J Cancer Biol Therap.

[9] Park CH, Han SE, Nam-Goong IS, Kim YI, Kim ES (2018). Combined effects of baicalein and docetaxel on apoptosis in 8505c anaplastic thyroid cancer cells via downregulation of the ERK and Akt/mTOR pathways. Endocrinol Metab.

[10] Lu X, Yang F, Chen D, Zhao Q, Chen D, Ping H, Xing N (2020). Quercetin reverses docetaxel resistance in prostate cancer via androgen receptor and PI3K/Akt signaling pathways. Int J Biol Sci.

[11] Lee LD, Mafura B, Lauscher JC, Seeliger H, Kreis ME, Gröne J (2014). Antiproliferative and apoptotic effects of telmisartan in human colon cancer cells. Oncol Lett.

[12] Funao K, Matsuyama M, Kawahito Y, Sano H, Chargui J, Touraine J-L, Nakatani T, Yoshimura R (2009). Telmisartan as a peroxisome proliferator-activated receptor-γ ligand is a new target in the treatment of human renal cell carcinoma. Mol Med Rep.

[13] Funao K, Matsuyama M, Kawahito Y, Sano H, Chargui J, Touraine JL, Nakatani T, Yoshimura R (2008). Telmisartan is a potent target for prevention and treatment in human prostate cancer. Oncol Rep.

[14] Kobara H, Fujihara S, Iwama H, Matsui T, Fujimori A, Chiyo T, Tingting S, Kobayashi N, Nishiyama N, Yachida T, Tadokoro T, Oura K, Tani J, Fujita K, Nomura T, Yoneyama H, Morishita A, Okano K, Suzuki Y, Mori H, Masaki T (2020). Antihypertensive drug telmisartan inhibits cell proliferation of gastrointestinal stromal tumor cells in vitro. Mol Med Rep.

[15] Abbasi A, Mostafavi-Pour Z, Amiri A, Keshavarzi F, Nejabat N, Ramezani F, Sardarian A, Zal F (2020). Chemoprevention of Prostate Cancer Cells by Vitamin C plus Quercetin: role of Nrf2 in Inducing Oxidative Stress. Nutr Cancer.

[16] Wu GS, Lu JJ, Guo JJ, Huang MQ, Gan L, Chen XP, Wang YT (2013). Synergistic anti-cancer activity of the combination of dihydroartemisinin and doxorubicin in breast cancer cells. Pharmacol Rep.

[17] Pfaffl MW (2001). A new mathematical model for relative quantification in real-time RT–PCR. Nucleic Acids Res.

[18] Chou TC (2006). Theoretical basis, experimental design, and computerized simulation of synergism and antagonism in drug combination studies. Pharmacol Rev.

[19] Elwakeel A, Soudan H, Eldoksh A, Shalaby M, Eldemellawy M, Ghareeb D, Abouseif M, Fayad A, Hassan M, Saeed H (2019). Implementation of the Chou-Talalay method for studying the in vitro pharmacodynamic interactions of binary and ternary drug combinations on MDA-MB-231 triple negative breast cancer cells. Synergy.

[20] Siegel RL, Miller KD, Fuchs HE, Jemal A (2021). Cancer statistics, 2021. CA Cancer J Clin.

[21] Mansour M, van Ginkel S, Dennis JC, Mason B, Elhussin I, Abbott K, Pondugula SR, Samuel T, Morrison E (2018). The combination of omega-3 stearidonic acid and docetaxel enhances cell death over docetaxel alone in human prostate cancer cells. J Cancer.

[22] Inamura S, Ito H, Taga M, Tsuchiyama K, Hoshino H, Kobayashi M, Yokoyama O (2019). Low-dose Docetaxel Enhanced the Anticancer Effect of Temsirolimus by Overcoming Autophagy in Prostate Cancer Cells. Anticancer Res.

[23] Matsuyama M, Funao K, Kuratsukuri K, Tanaka T, Kawahito Y, Sano H, Chargui J, Touraine J-L, Yoshimura N, Yoshimura R (2010). Telmisartan inhibits human urological cancer cell growth through early apoptosis. Exp Ther Med.

[24] Koyama N, Nishida Y, Ishii T, Yoshida T, Furukawa Y, Narahara H (2014). Telmisartan induces growth inhibition, DNA double-strand breaks and apoptosis in human endometrial cancer cells. PLoS One..

[25] Uemura H, Hasumi H, Kawahara T, Sugiura S, Miyoshi Y, Nakaigawa N, Teranishi Ji, Noguchi K, Ishiguro H, Kubota Y (2005). Pilot study of angiotensin II receptor blocker in advanced hormone-refractory prostate cancer. Int J Clin Oncol.

[26] Matsui T, Chiyo T, Kobara H, Fujihara S, Fujita K, Namima D, Nakahara M, Kobayashi N, Nishiyama N, Yachida T, Morishita A, Iwama H, Masaki T (2019). Telmisartan Inhibits Cell Proliferation and Tumor Growth of Esophageal Squamous Cell Carcinoma by Inducing S-Phase Arrest In Vitro and In Vivo. Int J Mol Sci..

[27] Fujita N, Fujita K, Iwama H, Kobara H, Fujihara S, Chiyo T, Namima D, Yamana H, Kono T, Takuma K, Hirata M, Kobayashi K, Kato K, Kamada H, Morishita A, Tsutsui K, Himoto T, Okano K, Suzuki Y, Masaki T (2020). Antihypertensive drug telmisartan suppresses the proliferation of gastric cancer cells in vitro and in vivo. Oncol Rep.

[28] Green R, Howell M, Khalil R, Nair R, Yan J, Foran E, Katiri S, Banerjee J, Singh M, Bharadwaj S, Mohapatra SS, Mohapatra S (2019). Actinomycin D and telmisartan combination targets Lung cancer Stem cells through the Wnt/Beta catenin pathway. Sci Rep.

[29] Zhang S, Wang Y (2018). Telmisartan inhibits NSCLC A549 cell proliferation and migration by regulating the PI3K/AKT signaling pathway. Oncol Lett.

[30] Tian H, Yang J, Xie Z, Liu J (2018). Gliquidone alleviates diabetic nephropathy by inhibiting notch/snail signaling pathway. Cell Physiol Biochem.

[31] Liu Y, Chen S, Liu J, Jin Y, Yu S, An R (2020). Telmisartan inhibits oxalate and calcium oxalate crystal-induced epithelial-mesenchymal transformation via PPAR-γ-AKT/STAT3/p38 MAPK-Snail pathway. Life Sci.

[32] Baygi ME, Soheili ZS, Essmann F, Deezagi A, Engers R, Goering W, Schulz WA (2010). Slug/SNAI2 regulates cell proliferation and invasiveness of metastatic prostate cancer cell lines. Tumor Biol.

